# Sarcopenic obesity assessed using dual energy X-ray absorptiometry (DXA) can predict cardiovascular disease in patients with type 2 diabetes: a retrospective observational study

**DOI:** 10.1186/s12933-018-0700-5

**Published:** 2018-04-10

**Authors:** Tatsuya Fukuda, Ryotaro Bouchi, Takato Takeuchi, Kazutaka Tsujimoto, Isao Minami, Takanobu Yoshimoto, Yoshihiro Ogawa

**Affiliations:** 1 Department of Molecular Endocrinology and Metabolism, Graduate School of Medical and Dental Sciences, Tokyo Medical and Dental University, 1-5-45 Yushima, Bunkyo-ku, Tokyo, 113-8510 Japan; 2 Department of Diabetes, Endocrinology and Metabolism, National Center for Global Health and Medicine, Tokyo, Japan; 3 Diabetes and Metabolism Information Center, National Center for Global Health and Medicine, Tokyo, Japan; 4 Department of Medicine and Bioregulatory Science, Graduate School of Medical Sciences, Kyushu University, Fukuoka, Japan; 5 Department of Molecular and Cellular Metabolism, Graduate School of Medical and Dental Sciences, Tokyo Medical and Dental University, Tokyo, Japan

**Keywords:** Sarcopenic obesity, Cardiovascular disease, Visceral adiposity, Type 2 diabetes, Dual-energy X-ray absorptiometry

## Abstract

**Background:**

Sarcopenic obesity, defined as reduced skeletal muscle mass and power with increased adiposity, was reported to be associated with cardiovascular disease risks in previous cross-sectional studies. Whole body dual-energy X-ray absorptiometry (DXA) can simultaneously evaluate both fat and muscle mass, therefore, whole body DXA may be suitable for the diagnosis of sarcopenic obesity. However, little is known regarding whether sarcopenic obesity determined using whole body DXA could predict incident cardiovascular disease (CVD). The aim of this study was to investigate the impact of sarcopenic obesity on incident CVD in patients with type 2 diabetes.

**Methods:**

A total of 716 Japanese patients (mean age 65 ± 13 years; 47.0% female) were enrolled. Android fat mass (kg), gynoid fat mass (kg), and skeletal muscle index (SMI) calculated as appendicular non-fat mass (kg) divided by height squared (m^2^), were measured using whole body DXA. Sarcopenic obesity was defined as the coexistence of low SMI and obesity determined by four patterns of obesity as follows: android to gynoid ratio (A/G ratio), android fat mass or percentage of body fat (%BF) was higher than the sex-specific median, or body mass index (BMI) was equal to or greater than 25 kg/m^2^. The study endpoint was the first occurrence or recurrence of CVD.

**Results:**

Over a median follow up of 2.6 years (IQR 2.1–3.2 years), 53 patients reached the endpoint. Sarcopenic obesity was significantly associated with incident CVD even after adjustment for the confounding variables, when using A/G ratio [hazard ratio (HR) 2.63, 95% CI 1.10–6.28, p = 0.030] and android fat mass (HR 2.57, 95% CI 1.01–6.54, p = 0.048) to define obesity, but not %BF (HR 1.67, 95% CI 0.69–4.02, p = 0.252), and BMI (HR 1.55, 95% CI 0.44–5.49, p = 0.496).

**Conclusions:**

The present data suggest that the whole body DXA is valuable in the diagnosis of sarcopenic obesity (high A/G ratio or android fat mass with low SMI) to determine the risk of CVD events in patients with type 2 diabetes. Meanwhile, sarcopenic obesity classified with low SMI, and high %BF or BMI was not associated with incident CVD.

**Electronic supplementary material:**

The online version of this article (10.1186/s12933-018-0700-5) contains supplementary material, which is available to authorized users.

## Background

Obesity is well known to be associated with various comorbidities such as type 2 diabetes, dyslipidemia, hypertension, and cardiovascular disease (CVD) [1]. In particular, visceral adiposity is reported to have greater impact on metabolic abnormalities [2], CVD [3], and mortality [4], compared with total adiposity as usually represented by body mass index (BMI). A recent large-scale prospective study revealed that visceral adiposity has been more useful to predict the risk of CVD than BMI in mostly non-diabetic subjects [5], and we showed that not BMI but visceral fat accumulation measured by dual bioelectrical impedance analyzer could predict incident CVD in patients with type 2 diabetes [6]. CVD is the leading cause of death in diabetic patients [7], therefore, accurate assessment of visceral fat accumulation is critical for assessing the risk of CVD, especially in patients with diabetes.

In addition to obesity, sarcopenia, defined as loss of skeletal muscle mass and strength with aging, has been reported to be associated with not only physical disability but also CVD. Ochi et al. [8] showed that sarcopenia is associated with increased arterial stiffness in middle-aged to elderly men, and Chin et al. [9] reported that sarcopenia was independently associated with the presence of CVD after adjusting for other cardiovascular risk factors in a large scale cross-sectional population-based study. Sarcopenia often co-occurs with obesity, the state called sarcopenic obesity, which contributes to significantly increased risks for physical disability and mobility impairment compared with sarcopenia or obesity alone [10, 11]. Furthermore, several cross-sectional studies demonstrated that individuals with sarcopenic obesity have a greater risk of hyperglycemia, hypertension, dyslipidemia, and insulin resistance than individuals without sarcopenic obesity [12–14]. Therefore, it is conceivable that individuals with sarcopenic obesity are at a high risk for incident CVD. Actually in the 5th Korean National Health and Nutrition Examination Survey, individuals with sarcopenic obesity defined by low muscle mass and high BMI had significantly high 10-year CVD risk determined using the Framingham risk model, whereas sarcopenic non-obese and non-sarcopenic obese individuals were not associated with an increased 10-year CVD risk [15]. However, there are very few observational studies that investigated the association between sarcopenic obesity evaluated based on direct measures of body fat and muscle mass, and incident CVD.

In recent years, visceral adiposity was reported to contribute to the progression of sarcopenia via physical inactivity, inflammatory cytokines such as interleukin-6 and tumor necrosis factor-α, and insulin resistance [16, 17]. Indeed, it is reported that patients with diabetes have an increased risk for the excessive loss of both skeletal muscle mass and strength in large scale longitudinal surveys [18, 19]. These findings presumably indicated that patients with diabetes have a higher risk of sarcopenic obesity, and sarcopenic obesity could partially account for the close relationship between diabetes and CVD. Nevertheless, little evidence was available to investigate the impact of sarcopenic obesity on incident CVD in patients with type 2 diabetes.

As for the direct measures of body fat distribution, computed tomography (CT) is considered the gold standard for abdominal fat quantification [20]. However, CT examination has some defects in clinical use, including exposure to radiation and high cost. Whole body dual-energy X-ray absorptiometry (DXA) can simultaneously evaluate both regional fat and muscle mass with much less radiation exposure than CT examination [21], and can accurately measure visceral fat as with CT examination [22]. Therefore, DXA may be suitable for the diagnosis of sarcopenic obesity. In addition, percentage of body fat (%BF) as indicator for total adiposity can be measured in the whole body DXA examination. Some previous studies were conducted using DXA often used %BF as obesity indicator in the examination of the association between sarcopenic obesity and cardiometabolic risks [11, 23, 24], however, few studies compared the predictive ability of each indicator for total and visceral adiposity obtained using the whole body DXA in examining the association between sarcopenic obesity and cardiometabolic risks or CVD.

Consequently, we carried out a retrospective observational study to examine whether sarcopenic obesity defined using the whole body DXA could predict incident CVD, and which indicator of obesity obtained from the whole body DXA is appropriate for diagnosis of sarcopenic obesity in prediction of CVD in patients with type 2 diabetes.

## Methods

### Subject

**Fig. 1:**
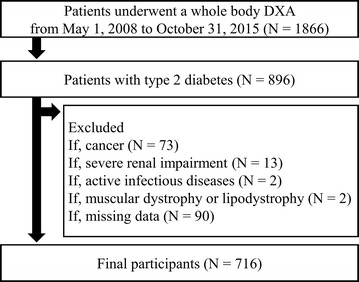
Study flow chart of participants

This retrospective, observational study included patients aged older than 20 years who regularly visited to Tokyo Medical and Dental University Hospital between 1 May 2008 and 31 October 2015. Patients were eligible if they were diagnosed with type 2 diabetes according to the criteria of the Japan Diabetes Society (JDS) [25], and underwent a whole body DXA during the study period. As shown in Fig. 1, 1866 patients had undergone a whole body DXA during the study period at our hospital, of whom 896 patients were diagnosed as type 2 diabetes. Exclusion criteria included renal impairment (estimated glomerular filtration rate (GFR) [eGFR] < 15 ml/min/1.73 m^2^ or undergoing renal replacement therapy), pregnancy, infectious diseases, muscular dystrophy, lipodystrophy and cancer. Finally, this study included 716 patients (Fig. 1). This study complies with the principles laid by Declaration of Helsinki and has been approved by the ethical committee of Tokyo Medical and Dental University (No. M2017-260).

### Clinical and biochemical analysis

Patient information about smoking status, medical history including the history of CVD, the severity of diabetic retinopathy, and medication were extracted from the medical records. Smoking was classified as non-smoker or current smoker. BMI was calculated based on the following formula: Bodyweight in kg divided by height in meters squared. Systolic and diastolic blood pressures (SBP and DBP) were measured after 5 min of seated rest, using an electronic sphygmomanometer (ES-H55, Terumo Inc., Tokyo, Japan). The measurement of HbA1c was performed using latex agglutination method. HbA1c values estimated as the JDS method was converted to the National Glycohemoglobin Standardization Program values [25]. GFR was estimated using the following equation for the Japanese, as proposed by the Japanese Society of Nephrology [26]; GFR = 194 × SCr^− 1.094^ × age^− 0.287^ [(if female) × 0.739], where SCr stands for serum creatinine in mg/dl, measured using an enzymatic method. Urinary albumin and creatinine concentrations were measured using turbidimetric immunoassay and enzymatic method, and the urinary albumin-to-creatinine ratio (ACR, mg/g) was calculated for the assessment of albuminuria in a spot urine sample.

### Body composition measuring by DXA

Body composition was measured using the whole body DXA (Lunar iDXA; GE Healthcare, Madison, Wisconsin, USA) by certified radiological technologists. The total fat mass and non-fat mass, percentage of body fat (%BF), and the fat masses of android and gynoid regions were measured as previously described [22, 27]. Skeletal muscle index (SMI) was calculated based on the following formula: Appendicular skeletal muscle mass (fat-free mass in the upper and lower extremities) in kg divided by height in meters squared. A/G ratio was the ratio of android divided by gynoid.

### Definition of sarcopenia, obesity, and sarcopenic obesity

Sarcopenia was defined as SMI less than 7.0 kg/m^2^ (in men) or 5.4 kg/m^2^ (in women) according to the criteria for Asians [28]. Studies have shown important relationships between A/G ratio and CVD risks including glycemic, lipid and blood pressure control in healthy subjects [29, 30]. In addition, we previously reported that A/G ratio is significantly and strongly correlated with visceral fat area (VFA) measured by CT both in male and female, and have a similar ability to VFA in the prediction of CVD risks in patients with type 2 diabetes [22]. Therefore, A/G ratio could be a valuable indicator for visceral adiposity. In addition to A/G ratio, several indicators of obesity are obtained in a whole body DXA such as android fat mass, and %BF. To evaluate the predictive ability of the indicators of obesity for incident CVD, obesity in the present study was defined in four ways as follows: A/G ratio was higher than the sex-specific median value in the present cohort (> 0.80 for men, > 0.62 for women), android fat mass was higher than the sex-specific median in the present cohort (> 2.16 kg for men, > 1.95 kg for women), and %BF was higher than the sex-specific median in the present cohort (> 31.8% for men, > 38.8% for women), or BMI was equal to or greater than 25 kg/m^2^ [31]. Participants were classified into four categories of body composition according to each indicator of obesity as follows; normal (participants without either sarcopenia or obesity), sarcopenia (with sarcopenia and without obesity), obesity (without sarcopenia and with obesity), and sarcopenic obesity (with both sarcopenia and obesity).

### Definition of cardiovascular disease

Incident CVD was defined as stroke, unstable angina, myocardial infarction, percutaneous coronary intervention, coronary bypass grafting, revascularization for carotid artery disease, angioplasty or major amputation due to peripheral arterial disease (PAD) or CVD death. The study endpoint was the first occurrence or recurrence of incident CVD.

### Statistical analysis

Statistical analysis was carried out using SPSS (version 21.0; IBM Corp, Armonk, New York, USA). Data are presented as the mean ± standard deviation (SD), or the median and interquartile range (IQR) according to the data distribution. Chi square test or Fisher’s exact test were used to compare categorical variables, and ANOVA followed by Tukey’s test for the post hoc analyses was used to compare the quantitative variables among patients with the four categories of body composition (normal, sarcopenia, obesity, and sarcopenic obesity). The hazard ratios (HRs) for the study endpoint of the categories of body composition were estimated using Cox proportional hazard models with normal category as the reference. In the multivariate model, the following variables were incorporated to the Cox regression analysis with a stepwise procedure: age, gender, duration of diabetes, history of CVD, smoking status, SBP, DBP, log-transformed ACR, eGFR, prevalence of proliferative diabetic retinopathy (PDR), triglycerides, high-density lipoprotein (HDL) cholesterol, low-density lipoprotein (LDL) cholesterol, HbA1c, aspartate transaminase (AST), alanine transaminase (ALT), gamma-glutamyl transpeptidase (γ-GTP), uric acid (UA), and the use of insulin, sulfonylureas, metformins, alpha-glucosidase inhibitors, glinides, thiazolidinediones (TZDs), dipeptidyl peptidase 4 (DPP4) inhibitors, sodium-glucose cotransporter 2 (SGLT-2) inhibitors, glucagon-like peptide-1 receptors agonists (GLP1-RAs), calcium channel blockers (CCBs), angiotensin converting enzyme inhibitors (ACEIs) or angiotensin receptor blockers (ARBs), alpha blockers, beta blockers, diuretics, statins, fibrates, ezetimibe, eicosapentaenoic acids (EPAs), UA lowering agents and anti-platelet agents at baseline. Next, we created another multivariate model in which gender was entered in addition to the covariates selected in the multivariate model because male sex is one of the important risk factors for incident CVD and there is a clear difference in the body composition between men and women (gender-adjusted multivariate model). In addition to A/G ratio, we further calculated HRs of sarcopenic obesity when android fat mass, %BF or BMI was used for the classification of obesity, respectively. P-values less than 0.05 were considered to be statistically significant.

## Results

### Clinical characteristics of the study population

Among the participants, 187 (26.1%), 171 (23.9%), 275 (38.4%) and 83 (11.6%) patients were classified as normal, sarcopenia, obesity and sarcopenic obesity, respectively, and Table 1 shows the clinical characteristics at baseline in the four categories of body composition, when using A/G ratio for the classification of obesity. The age and the proportion of men were significantly higher in sarcopenia and sarcopenic obesity patients than normal and obesity patients. The duration of diabetes was significantly longer in sarcopenic obesity patients than that in patients of the other categories. Moreover, DBP, triglycerides, AST, ALT, γ-GTP and UA were significantly higher in obesity patients than in patients of the other categories. The HDL cholesterol level was lower in both obesity and sarcopenic obesity patients than normal and sarcopenia patients. There were no significant differences in the proportion of current smoker, history of CVD, SBP, log-transformed ACR, eGFR, prevalence of PDR, and HbA1c. Medications in the four categories of body composition, when using A/G ratio for the classification of obesity, were listed in Table 2. There were significant differences in the frequency of receiving metformins, DPP4 inhibitors, ARBs, CCBs, Diuretics, statins and UA lowering agents among the four categories of body composition.

**Table 1:** Clinical characteristics at baseline in the four categories of body composition classified using A/G ratio

	Normal (n = 187)	Sarcopenia (n = 171)	Obesity (n = 275)	Sarcopenic Obesity (n = 83)	p value*
Age (years)	62 ± 14	69 ± 11^†^	63 ± 12	70 ± 10^†^	< 0.001
Gender (% male)	39.6	67.3	46.5	75.9	< 0.001
Body fat (%)	33.7 ± 7.2	28.1 ± 7.9^†^	38.8 ± 6.4^†^	35.3 ± 5.3	< 0.001
Android fat mass (kg)	1.8 (1.4–2.3)	1.2 (0.8–1.6)^†^	2.8 (2.3–3.7)^†^	2.1 (2.1–3.5)^†^	< 0.001
Gynoid fat mass (kg)	3.1 (2.6–3.7)	2.1 (1.6–2.7)^†^	3.4 (2.8–4.3)^†^	2.5 (2.5–4.0)^†^	< 0.001
A/G ratio	0.57 ± 0.14	0.53 ± 0.17	0.86 ± 0.15^†^	0.87 ± 0.12^†^	< 0.001
SMI (kg/m^2^)	6.86 ± 1.05	5.70 ± 0.88^†^	7.41 ± 1.15^†^	6.00 ± 0.79^†^	< 0.001
Body mass index (kg/m^2^)	25.3 ± 3.8	20.8 ± 2.9^†^	29.1 ± 5.3^†^	23.7 ± 3.0^†^	< 0.001
Duration of diabetes (years)	10.2 ± 9.6	11.4 ± 10.9	10.7 ± 8.8	15.3 ± 13.1^†^	0.001
History of CVD (%)	19	22	21	33	0.092
Current smoker (%)	12	16	11	22	0.083
SBP (mmHg)	131 ± 22	127 ± 18	133 ± 18	130 ± 19	0.072
DBP (mmHg)	77 ± 14	73 ± 12	78 ± 12^†^	76 ± 13	0.001
Log ACR (mg/gCr)	1.69 ± 0.76	1.76 ± 0.75	1.75 ± 0.78	1.85 ± 0.77	0.497
eGFR (ml/min/1.73 m^2^)	73.8 ± 22.2	75.5 ± 29.8	69.5 ± 22.7	70.2 ± 25.9	0.054
PDR (%)	9	7	13	10	0.197
HbA1c (%)	8.2 ± 2.0	8.1 ± 2.1	8.3 ± 1.7	8.6 ± 1.8	0.266
HbA1c (mmol/mol)	66.2 ± 21.2	64.9 ± 22.3	67.6 ± 18.4	70.0 ± 20.0	0.266
Triglycerides (mmol/l)	1.32 (0.85–1.80)	1.12 (0.82–1.76)	1.56 (1.10–2.21)^†^	1.29 (1.08–2.11)	< 0.001
HDL cholesterol (mmol/l)	1.50 ± 0.50	1.59 ± 0.53	1.33 ± 0.34^†^	1.31 ± 0.41^†^	< 0.001
LDL cholesterol (mmol/l)	2.80 ± 0.94	2.74 ± 0.78	2.90 ± 0.82	2.89 ± 0.97	0.233
AST (U/l)	21 (17–27)	21 (18–27)	24 (19–33)^†^	21 (19–31)	< 0.001
ALT (U/l)	19 (14–28)	17 (13–23)	25 (18–42)^†^	18 (16–38)	< 0.001
γ-GTP (U/l)	25 (18–48)	30 (20–48)	34 (22–59)^†^	33 (22–59)	0.001
UA (μmol/l)	297 ± 90	287 ± 80	335 ± 86^†^	325 ± 90	< 0.001

ACR, albumin-to-creatinine ratio; A/G, android to gynoid fat; ALT, *alanine transaminase*; AST, aspartate transaminase; CVD, cardiovascular disease; DBP, diastolic blood pressure; eGFR, estimated glomerular filtration ratio; GTP, *glutamyl transpeptidase*; HDL, high-density lipoprotein; LDL, low-density lipoprotein; PDR, proliferative diabetic retinopathy; SBP, systolic blood pressure; SMI, skeletal muscle index; UA, uric acid

* p value for difference among the four groups in percepts (Chi square test or Fisher’s exact test) or means (ANOVA)

^†^p < 0.05 vs normal patients by Tukey’s test

**Table 2:** Medication at baseline in the four categories of body composition classified using A/G ratio

	Normal (n = 187)	Sarcopenia (n = 171)	Obesity (n = 275)	Sarcopenic obesity (n = 83)	p value*
Insulin (%)	34	39	36	39	0.756
Sulfonylureas (%)	20	21	24	21	0.652
Metformins (%)	24	15	43	29	< 0.001
Alpha-GIs (%)	16	16	10	12	0.146
Glinides (%)	5	4	3	5	0.704
TZDs (%)	9	3	8	5	0.068
DPP4 inhibitors (%)	27	30	39	46	0.005
SGLT2 inhibitors (%)	1	0	1	1	0.631
GLP1-RAs (%)	2	2	4	1	0.581
ACEIs (%)	5	2	3	5	0.396
ARBs (%)	41	32	56	50	< 0.001
CCBs (%)	32	28	46	43	< 0.001
Alpha blockers (%)	1	1	3	0	0.122
Beta blockers (%)	11	8	11	18	0.129
Diuretics (%)	10	10	8	12	0.790
Statins (%)	47	31	54	40	< 0.001
Fibrates (%)	5	2	2	2	0.103
Ezetimib (%)	5	2	2	0	0.082
EPAs (%)	3	2	3	5	0.774
UA lowering agents (%)	8	8	15	13	0.029
Anti-platelet agents (%)	18	22	26	33	0.050

ACEIs, angiotensin converting enzyme inhibitors; ARBs, angiotensin receptor blockers, CCBs, calcium channel blockers; DPP4, dipeptidyl peptidase 4; EPA, eicosapentaenoic acid; GIs, glycosidase inhibitors; GLP1-RA, glucagon-like peptide-1 receptors agonist; SGLT2, sodium-glucose cotransporter 2; TZDs, *thiazolidinediones*; UA, uric acid

* Chi square test or Fisher’s exact test

The baseline characteristics and medications in the four categories of body composition classified according to each indicator of obesity other than A/G ratio are shown in Additional file 1: Tables S1–S6. Additional file 1: Table S1 shows the baseline characteristics when using android fat mass for the classification of obesity. Sarcopenia patients were older, and obesity patients were younger than normal patients. The proportion of men were significantly higher in both sarcopenia and sarcopenic obesity patients than normal and obesity patients. The duration of diabetes was significantly longer in sarcopenic obesity patients than that in patients of the other categories. SBP, triglycerides, AST, ALT, and UA were significantly higher in obesity patients than in patients of the other categories. DBP and γ-GTP were higher and HDL cholesterol level was lower in both obesity and sarcopenic obesity patients than normal and sarcopenia patients. There were significant differences in the frequency of receiving metformins, TZDs, ARBs, CCBs, Beta blockers, statins, ezetimib and UA lowering agents among the four categories of body composition, when using android fat mass for the classification of obesity (Additional file 1: Table S2). Additional file 1: Table S3 shows the baseline characteristics when using %BF for the classification of obesity. The age and the proportion of men were significantly higher in both sarcopenia and sarcopenic obesity patients than normal and obesity patients. The duration of diabetes was significantly longer in sarcopenic obesity patients than that in patients of the other categories. SBP, DBP, triglycerides, AST, ALT, γ-GTP and UA were significantly higher in obesity patients than in patients of the other categories. HDL cholesterol level was lower in obesity and sarcopenic obesity patients than normal and sarcopenia patients. There were significant differences in the frequency of receiving metformins, TZDs, ARBs, CCBs, Beta blockers, statins and ezetimib among the four categories of body composition, when using %BF for the classification of obesity (Additional file 1: Table S4). Additional file 1: Table S5 shows the baseline characteristics when using BMI for the classification of obesity. The age and the proportion of men were significantly higher in both sarcopenia and sarcopenic obesity patients than normal and obesity patients. The duration of diabetes was significantly longer in sarcopenic obesity patients than that in patients of the other categories. SBP, DBP, triglycerides and UA were significantly higher, and HDL cholesterol level was lower in obesity patients than in patients of the other categories. ALT was higher in obesity and sarcopenic obesity patients than normal and sarcopenia patients. AST was higher in sarcopenic obesity patients than that in patients of the other categories. There were significant differences in the frequency of receiving metformins, TZDs, DPP4 inhibitors, ARBs, CCBs, Beta blockers, statins, UA lowering agents and anti-platelet agents among the four categories of body composition, when using BMI for the classification of obesity (Additional file 1: Table S6).

### Prediction ability for incident CVD of sarcopenic obesity assessed by both A/G ratio and SMI

During the median follow-up period of 2.6 years (IQR 2.1–3.2 years), 53 patients reached the end-point including 16 events of stroke, 26 events of coronary artery disease, 3 events of carotid artery disease, 2 events of PAD and 6 events of CVD death. Table 3 shows HRs for the endpoint in the categories of body composition classified according to A/G ratio and SMI. In the univariate model, sarcopenic obesity was significantly associated with increased risk of CVD event, with a hazard ratio (HR) of 4.50 (95% CI 1.93–10.46, p = 0.006). The association between sarcopenic obesity and CVD event remained significant (HR 2.63, 95% CI 1.10–6.28, p = 0.030), even after the adjustment for covariates including HDL cholesterol (HR 0.90, 95% CI 0.94–0.99, p = 0.002), HbA1c (HR 1.28, 95% CI 1.09–1.49, p = 0.002), eGFR (HR 0.97, 95% CI 0.96–0.98, p < 0.001), the use of ACEIs or ARBs (HR 2.07, 95% CI 1.11–3.86, p = 0.022), the use of DPP4 inhibitors (HR 0.46, 95% CI 0.23–0.91, p = 0.025), and history of CVD (HR 2.58, 95% CI 1.57–5.18, p = 0.001) which were selected as significant covariates by stepwise procedure (multivariate model). In the gender-adjusted multivariate model, the association between sarcopenic obesity and the end-point was slightly attenuated after further adjustment for gender (HR 2.14, 95% CI 0.88–5.21, p = 0.093). On the other hand, sarcopenia (HR 1.43, 95% CI 0.58–3.52, p = 0.439 in univariate mode, HR 1.88, 95% CI 0.75–4.74, p = 0.179 in multivariate model, and HR 1.46, 95% CI 0.57–3.74, p = 0.429 in gender-adjusted multivariate model), and Obesity (HR 1.56, 95% CI 0.71–3.43, p = 0.269 in univariate model, HR 0.98, 95% CI 0.44–2.21, p = 0.969 in multivariate model, and HR 0.94, 95% CI 0.42–2.10, p = 0.870 in gender-adjusted multivariate model) were consistently not significantly associated with CVD event in all models.

**Table 3:** Hazard ratios of incident cardiovascular disease in the categories of body composition classified using A/G ratio

	HR	(95% CI)	p value
Univariate model			
Normal	1.00 (reference)	–	–
Sarcopenia	1.43	(0.58–3.52)	0.439
Obesity	1.56	(0.71–3.43)	0.269
Sarcopenic obesity	4.50	(1.93–10.46)	0.006
Multivariate model			
Normal	1.00 (reference)	–	–
Sarcopenia	1.88	(0.75–4.74)	0.179
Obesity	0.98	(0.44–2.21)	0.969
Sarcopenic obesity	2.63	(1.10–6.28)	0.030
HDL cholesterol (mmol/l)	0.21	(0.08–0.56)	0.002
HbA1c (%)	1.28	(1.09–1.49)	0.002
eGFR (ml/min/1.73 m^2^)	0.97	(0.96–0.98)	< 0.001
ACEIs or ARBs	2.07	(1.11–3.86)	0.022
DPP4 inhibitors	0.46	(0.23–0.91)	0.025
History of CVD	2.85	(1.57–5.18)	0.001
Gender-adjusted multivariate model			
Normal	1.00 (reference)	–	–
Sarcopenia	1.46	(0.57–3.74)	0.429
Obesity	0.94	(0.42–2.10)	0.870
Sarcopenic obesity	2.14	(0.88–5.21)	0.093
HDL cholesterol (mmol/l)	0.23	(0.09–0.62)	0.003
HbA1c (%)	1.28	(1.10–1.49)	0.001
eGFR (ml/min/1.73 m^2^)	0.97	(0.96–0.96)	< 0.001
ACEIs or ARBs	2.18	(1.17–4.10)	0.014
DPP4 inhibitors	0.46	(0.23–0.88)	0.020
History of CVD	2.65	(1.44–4.87)	0.001
Gender (male = 1)	1.67	(0.87–3.20)	0.126

ACEIs, angiotensin converting enzyme inhibitors; ARBs, angiotensin receptor blockers; CVD, cardiovascular disease; DPP4, dipeptidyl peptidase 4, CI, confidence interval; eGFR, estimated glomerular filtration rate; HDL, high-density lipoprotein; HR, hazard ratio

### Prediction ability for incident CVD of sarcopenic obesity classified according to the indicators of obesity other than A/G ratio and SMI

**Fig. 2:**
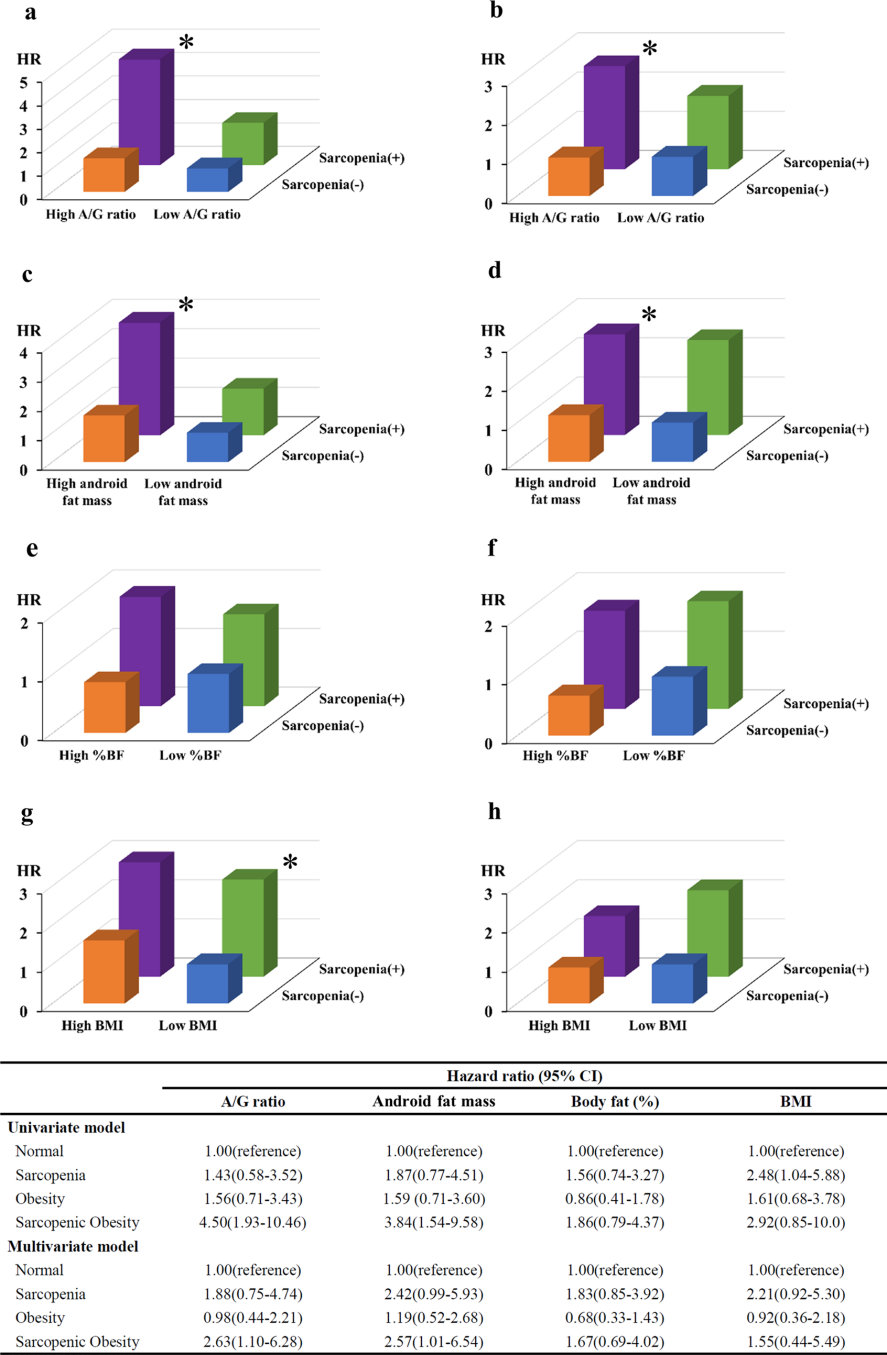
Hazard ratio for incident cardiovascular disease (CVD) in patients with type 2 diabetes classified as normal (blue), sarcopenia (green), obesity (orange), and sarcopenic obesity (purple) according to android to gynoid ratio (A/G ratio) (**a** univariate model; **b** multivariate model), android fat mass (**c** univariate model; **d** multivariate model), percentage of body fat (%BF) (**e** univariate model; **f** multivariate model), or body mass index (BMI) (**g** univariate model; **h** multivariate model), respectively. The multivariate models included high-density lipoprotein cholesterol, HbA1c, estimated glomerular filtration ratio, the use of angiotensin converting enzyme inhibitors or angiotensin receptor blockers, the use of dipeptidyl peptidase 4 inhibitors, and history of CVD as covariates. *p < 0.05 vs patients classified as normal by the Cox regression analysis

Next, we created additional statistical models in which whether sarcopenic obesity could predict the endpoint in each case android fat mass, %BF or BMI were selected instead of A/G ratio for the classification of body composition. Figure 2 shows HRs for the endpoint in the categories of body composition classified according to the A/G ratio in univariate (Fig. 2a) and multivariate (Fig. 2b) models, android fat mass in univariate (Fig. 2c) and multivariate (Fig. 2d) models, %BF in univariate (Fig. 2e) and multivariate (Fig. 2f) models, or BMI in univariate (Fig. 2g) and multivariate (Fig. 2h) models. The multivariate models were adjusted for independent variables including HDL cholesterol, HbA1c, eGFR, the use of ACEIs or ARBs, the use of DPP4 inhibitors, and history of CVD, all of which were identified as significant predictors for the end point in each model by a stepwise procedure. Sarcopenic obesity classified according to A/G ratio (HR 2.63, 95% CI 1.10–6.28, p = 0.030) or android (HR 2.57, 95% CI 1.01–6.54, p = 0.048) was significantly associated with the endpoint, whereas sarcopenic obesity classified according to %BF (HR 1.67, 95% CI 0.69–4.02, p = 0.252) and BMI (HR 1.55, 95% CI 0.44–5.49, p = 0.496) was not significantly associated with the endpoint in the multivariate models.

## Discussion

The present study firstly demonstrated that sarcopenic obesity assessed by the whole body DXA is an independent risk factor for CVD event in patients with type 2 diabetes, when using A/G ratio or android fat mass to determine obesity. The impact of sarcopenic obesity remained statistically significant after addition of well-known cardiovascular risk factors including HDL cholesterol, HbA1c, eGFR and history of CVD in the multivariate models. On the other hand, sarcopenic obesity classified according to markers for total adiposity (%BF or BMI) was not associated with CVD event.

### The association between sarcopenic obesity and cardiovascular disease

Besides obesity, sarcopenia was reported to be associated with an increased risk for CVD. A recent large-scale population-based study revealed that sarcopenic subjects have increased CVD risks compared to the non-sarcopenic subjects [32]. Furthermore, the study demonstrated that non-sarcopenic subjects with higher diet-induced acid load, which was independently associated with increased risk of CVD, had lower CVD risk than sarcopenic subjects with lower diet-induced acid load. Hence, it is conceivable that sarcopenia could be associated with CVD risk as well as obesity, and individuals with sarcopenic obesity had a significantly higher risk of CVD. Currently, a large number of studies have been carried out to investigate the association sarcopenic obesity and cardiovascular risk factors. Several cross-sectional studies from Korea revealed that sarcopenic obesity is associated with an increased risk for hypertension [33], dyslipidemia [34], insulin resistance [13], and metabolic syndrome [35] compared with sarcopenia or obesity alone. Furthermore, in a large cross-sectional analysis of 14,528 subjects from the National Health and Nutrition Examination Survey III reported that individuals with sarcopenic obesity had the highest risk of adverse glucose metabolism [12]. Nevertheless, the longitudinal association between sarcopenic obesity and CVD event has been surveyed in only a few studies. In the prospective survey of 3366 community-dwelling older men and women, sarcopenic obesity evaluated using waist circumference (WC) and muscle strength was associated with the highest risk of CVD event [36]. On the other hand, the prospective survey of 4252 older men from the British Regional Heart Study showed that the risk of CVD events was not significantly greater in the sarcopenic obese group defined using WC and midarm muscle circumference [37]. The inconsistencies of the two longitudinal studies in the associations between sarcopenic obesity and CVD event may be partially explained by the heterogeneity in the definition and classification of sarcopenic obesity. Indeed, Kwon YN reported that the prevalence of SO varies from 0 to 48% in the previous studies depending on the background of the studied population, parameters, and cut-off values [38]. Therefore, definition of sarcopenic obesity which is useful for predicting incident CVD needs to be established. Regarding obesity, WC is generally used to evaluate the visceral fat mass as an indirect measure, however, WC was reported to be impossible to differentiate between visceral and subcutaneous fat depots [39]. Actually, Mahabadi et al. reported that visceral fat tissue measured by CT is significantly associated with CVD even after adjusted for the covariates including BMI and WC, indicating the inaccuracy of WC for the measurement of visceral adiposity to estimate future CVD events [40]. In addition, we previously demonstrated that the ratio of abdominal visceral-to-subcutaneous fat area measured by a dual bioelectrical impedance analyzer independently of cardiovascular risk factors can predict CVD events in patients with type 2 diabetes [6]. To overcome the defect of WC, a body shape index (ABSI) has been recently developed as a new anthropometric parameter based on WC, BMI and height for the assessment of visceral adiposity [41]. ABSI was reported to be able to better predict mortality than WC, and we previously demonstrated that not WC but ABSI is independently associated with arterial stiffness evaluated using brachial-ankle pulse wave velocity (baPWV) in patients with type 2 diabetes [42]. Furthermore, it is reported that ABSI is inversely correlated with muscle mass and may be useful for the diagnosis of sarcopenic obesity [43, 44]. However, it is uncertain whether sarcopenic obesity diagnosed using ABSI could be associated with increased cardiometabolic risks. Given these findings, it is conceivable that direct measurement of visceral fat may be much more relevant for the diagnosis of sarcopenic obesity. However, no data have been available so far on the longitudinal associations of the sarcopenic obesity assessed by the direct measurement of visceral fat with CVD events in patients with diabetes. In the present retrospective observational study, we have provided evidence for the first time that sarcopenic obesity defined by markers for visceral fat (A/G ratio or android fat mass) and SMI all of which can be obtained by the whole body DXA is useful for predicting CVD events in patients with type 2 diabetes.

### Predictive ability of each indicator for total and visceral obesity in examining the relationship between sarcopenic obesity and CVD

In this study, BMI and %BF were used as measures of total adiposity, and A/G ratio and android fat mass were used as measures of visceral adiposity. Since DXA can simultaneously evaluate both regional fat and muscle mass, DXA was used in many studies to evaluate sarcopenia obesity with various indicators similarly to our study. Of the various indicators, %BF was widely used in previous reports [11, 23, 24, 45]. However, a recent study demonstrated that abdominal fat mass is strongly associated with CVD risk factors in the present study compared to total fat mass in whole body DXA examination [46]. In addition, although there is no report investigating the longitudinal association of CVD with sarcopenic obesity which is diagnosed based on the indicators obtained by DXA, a population-based prospective cohort study of 1485 participants reported that sarcopenic obesity diagnosed using SMI (measured by a bioelectrical impedance analysis) and index of abdominal obesity (high triglycerides with high waist circumference) have a high cardiovascular mortality risk [47]. This association of sarcopenic obesity with cardiovascular mortality remains unchanged after adjusted for BMI. These findings supported the hypothesis that indicators of visceral adiposity were more suitable for the diagnosis of sarcopenic obesity with the whole body DXA in prediction for CVD. In consistent with these findings, we clearly showed that indicators of visceral adiposity (i.e., A/G ratio and android fat mass) are more appropriate for the classification of sarcopenic obesity than indicators of total adiposity (i.e., %BF and BMI) in patients with type 2 diabetes.

## Limitations

The present study has several limitations. First, the follow-up period was relatively short, and the number of CVD events was small. Second, the characteristics of patients in the present study were homogenous due to hospital-based design, therefore, generalization of our findings might be limited. Third, we were unable to obtain the information on muscle strength such as handgrip power. It has been reported that muscular strength is useful for predicting incident CVD in the diagnosis of sarcopenia obesity. Therefore, further studies are needed to investigate the association between sarcopenic obesity which is diagnosed using both muscle mass and strength and CVD. Fourth, we did not assess the change in body composition in the present study. As the body composition changes with aging or weight gain or loss, there is a possibility that the change of body composition affects the end point. Fifth, we were unable to carry out the sensitivity analysis by gender. It is well known that men store a greater amount of fat in the visceral depot. In addition, it is reported that there are gender differences in the prevalence of CVD and the impact of risk factors including obesity on incident CVD [48, 49]. Therefore, the sensitivity analysis by gender should be done to evaluate whether sarcopenic obesity could be associated with incident CVD in both gender. However, we could not develop valid statistical models of each gender because of the small sample size and relatively small number of female patients who reached the end-point (16 female patients reached the end-point). Finally, it is to be elucidated whether sarcopenic obesity diagnosed using A/G ratio or android measured with DXA could be associated with CVD in non-diabetic populations.

## Conclusions

The present data suggest that the whole body DXA is valuable in the diagnosis of sarcopenic obesity to determine the risk of CVD events when sarcopenic obesity was defined by low SMI and high visceral adiposity (A/G ratio or android fat mass) in patients with type 2 diabetes. Meanwhile, sarcopenic obesity classified with low SMI and high total adiposity (%BF or BMI) was not associated with an increased risk of CVD events.

## Additional file

**Additional file 1.** Clinical characteristics and medications at baseline in the four categories of body composition (normal, sarcopenia, obesity, and sarcopenic obesity) classified using android fat mass, percent of body fat and body mass index. The baseline characteristics and medications in the four categories of body composition classified according to each indicator of obesity other than A/G ratio are shown in Tables S1–S6. **Tables S1, S3, S5.** The baseline characteristics when using android fat mass, percent of body fat, and body mass index for the classification of obesity, respectively. **Tables S2, S4, S6.** The medication when using android fat mass, percent of body fat, and body mass index for the classification of obesity, respectively.

## Abbreviations

ACEIs: angiotensin converting enzyme inhibitors
ACR: albumin-to-creatinine ratio
A/G: android to gynoid fat
ALT: alanine transaminase
ARBs: angiotensin receptor blockers
AST: aspartate transaminase
%BF: percentage of body fat
BMI: body mass index
CCBs: calcium channel blockers
CT: computed tomography
CVD: cardiovascular disease
DBP: diastolic blood pressure
DPP4: dipeptidyl peptidase 4
eGFR: estimated glomerular filtration ratio
DXA: dual-energy X-ray absorptiometry
EPA: eicosapentaenoic acid
GIs: glycosidase inhibitors
GLP1-RA: glucagon-like peptide-1 receptors agonist
GTP: glutamyl transpeptidase
HDL: high-density lipoprotein
HRs: hazard ratios
IQR: interquartile range
JDS: Japan Diabetes Society
LDL: low-density lipoprotein
PDR: proliferative diabetic retinopathy
SBP: systolic blood pressure
SD: standard deviation
SGLT2: sodium-glucose cotransporter 2
SMI: skeletal muscle index
TZDs: thiazolidinediones
UA: uric acid
VFA: visceral fat area
WC: waist circumference
γ-GTP: gamma-glutamyl transpeptidase
