# Supplementary material for: Sarcopenic obesity assessed using dual energy X-ray absorptiometry (DXA) can predict cardiovascular disease in patients with type 2 diabetes: a retrospective observational study
Source: Cardiovasc Diabetol. 2018 Apr 10;17:55. doi: 10.1186/s12933-018-0700-5 (PMC5891961; doi:10.1186/s12933-018-0700-5)
Supplement: Supplementary file 1 — Additional file 1. Clinical characteristics and medications at baseline in the four categories of body composition (normal, sarcopenia, obesity, and sarcopenic obesity) classified using android fat mass, percent of body fat and body mass index. The baseline characteristics and medications in the four categories of body composition classified according to each indicator of obesity other than A/G ratio are shown in Tables S1–S6. Tables S1, S3, S5. The baseline characteristics when using android fat mass, percent of body fat, and body mass index for the classification of obesity, respectively. Tables S2, S4, S6. The medication when using android fat mass, percent of body fat, and body mass index for the classification of obesity, respectively. [file 12933_2018_700_MOESM1_ESM.docx]

**Table S1. Clinical characteristics at baseline in the four categories of body composition classified using android fat mass**

|  | **Normal**  **(n = 161)** | **Sarcopenia**  **(n = 197)** | **Obesity**  **(n = 301)** | **Sarcopenic Obesity**  **(n = 57)** | ***P value** |
| --- | --- | --- | --- | --- | --- |
| Age (years) | 65 ± 12 | 70 ± 11^†^ | 61 ± 12^†^ | 67 ± 10 | < 0.001 |
| Gender (%male) | 37.9 | 65.5 | 46.8 | 86.0 | < 0.001 |
| Body fat (%) | 31.0 ± 5.9 | 28.1 ± 7.2^†^ | 39.8 ± 5.8^†^ | 38.3 ± 4.4^†^ | < 0.001 |
| Android fat mass (kg) | 1.6 (1.3-1.8) | 1.3 (0.8-1.6) ^†^ | 2.9 (2.4-3.7) ^†^ | 2.7 (2.3-3.1) ^†^ | < 0.001 |
| Gynoid fat mass (kg) | 2.6 (2.1-2.9) | 2.1 (1.6-2.5) ^†^ | 3.7 (3.2-4.5) ^†^ | 3.1 (2.7-3.7) ^†^ | < 0.001 |
| A/G ratio | 0.60 ± 0.19 | 0.58 ± 0.20 | 0.82 ± 0.17^†^ | 0.87 ± 0.13^†^ | < 0.001 |
| SMI (kg/m^2^) | 6.66 ± 0.89 | 5.69 ± 0.87^†^ | 7.48 ± 1.16^†^ | 6.19 ± 0.73^†^ | < 0.001 |
| Body mass index (kg/m^2^) | 23.7 ± 2.7 | 20.7 ± 2.6^†^ | 29.6 ± 4.9^†^ | 25.2 ± 2.9^†^ | < 0.001 |
| Duration of diabetes (years) | 11.3 ± 10.1 | 12.1 ± 11.5 | 10.1 ± 8.6 | 14.4 ± 12.7^†^ | 0.011 |
| History of CVD (%) | 17 | 23 | 22 | 33 | 0.071 |
| Current smoker (%) | 11 | 16 | 12 | 23 | 0.086 |
| SBP (mmHg) | 128 ± 20 | 127 ± 19 | 135 ± 19^†^ | 131 ± 17 | < 0.001 |
| DBP (mmHg) | 73 ± 12 | 73 ± 12 | 79 ± 13^†^ | 79 ± 13^†^ | < 0.001 |
| Log ACR (mg/gCr) | 1.67 ± 0.75 | 1.79 ± 0.76 | 1.76 ± 0.79 | 1.77 ± 0.77 | 0.541 |
| eGFR (ml/min/1.73m^2^) | 74.2 ± 22.3 | 74.5 ± 29.1 | 69.5 ± 22.4 | 71.2 ± 27.0 | 0.105 |
| PDR (%) | 11 | 7 | 12 | 12 | 0.251 |
| HbA1c (%) | 8.2 ± 2.0 | 8.2 ± 2.1 | 8.3 ± 1.7 | 8.4 ± 1.7 | 0.814 |
| HbA1c (mmol/mol) | 66.1 ± 26.2 | 65.9 ± 21.7 | 67.2 ± 18.6 | 68.3 ± 18.1 | 0.814 |
| Triglycerides (mmol/l) | 1.31 (0.84-1.89) | 1.12 (0.82-1.65) | 1.57 (1.10-2.18) ^†^ | 1.40 (1.08-2.04) | < 0.001 |
| HDL cholesterol (mmol/l) | 1.48 ± 0.51 | 1.54 ± 0.52 | 1.36 ± 0.36^†^ | 1.37 ± 0.47^†^ | < 0.001 |
| LDL cholesterol (mmol/l) | 2.86 ± 0.96 | 2.79 ± 0.80 | 2.87 ± 0.87 | 2.80 ± 0.99 | 0.742 |
| AST (U/l) | 21 (18-25) | 21 (18-26) | 24 (19-32) ^†^ | 23 (19-31) | < 0.001 |
| ALT (U/l) | 19 (14-28) | 16 (12-23) | 26 (19-42) ^†^ | 22 (18-40) | < 0.001 |
| γ-GTP (U/l) | 24 (17-48) | 29 (18-47) | 34 (21-59) ^†^ | 36 (24-63) ^†^ | 0.001 |
| UA (μmol/L) | 289 ± 84 | 294 ± 85 | 336 ± 87^†^ | 318 ± 81 | < 0.001 |
| **Abbreviations:** ACR, albumin-to-creatinine ratio; A/G, android to gynoid fat; ALT, alanine transaminase; AST, aspartate transaminase; CVD, cardiovascular disease; DBP, diastolic blood pressure; eGFR, estimated glomerular filtration ratio; GTP, glutamyl transpeptidase; HDL, high-density lipoprotein; LDL, low-density lipoprotein; PDR, proliferative diabetic retinopathy; SBP, systolic blood pressure; SMI, skeletal muscle index; UA, uric acid.  *P Value for difference among the four groups in percepts (Chi-square test or Fisher's exact test) or means (ANOVA).  †P <0.05 vs Normal patients by Tukey's test. | | | | | |

**Table S2. Medication at baseline in the four categories of body composition classified using android fat mass**

|  | **Normal**  **(n = 161)** | **Sarcopenia**  **(n = 197)** | **Obesity**  **(n = 301)** | **Sarcopenic Obesity**  **(n = 57)** | ***P value** |
| --- | --- | --- | --- | --- | --- |
| Insulin (%) | 39 | 38 | 33 | 42 | 0.368 |
| Sulfonylureas (%) | 17 | 22 | 25 | 16 | 0.161 |
| Metoformins (%) | 20 | 15 | 43 | 37 | <0.001 |
| Alpha-GIs (%) | 12 | 16 | 12 | 11 | 0.538 |
| Glinides (%) | 4 | 4 | 3 | 4 | 0.946 |
| TZDs (%) | 7 | 3 | 10 | 5 | 0.039 |
| DPP4 inhibitors (%) | 29 | 33 | 37 | 44 | 0.148 |
| SGLT2 inhibitors (%) | 1 | 0 | 1 | 2 | 0.450 |
| GLP1-RAs (%) | 1 | 2 | 4 | 2 | 0.285 |
| ACEIs (%) | 2 | 3 | 5 | 4 | 0.529 |
| ARBs (%) | 40 | 34 | 55 | 53 | <0.001 |
| CCBs (%) | 34 | 28 | 44 | 49 | <0.001 |
| Alpha blockers (%) | 2 | 1 | 3 | 0 | 0.398 |
| Beta blockers (%) | 9 | 8 | 12 | 25 | 0.003 |
| Diuretics (%) | 7 | 8 | 10 | 18 | 0.134 |
| Statins (%) | 45 | 27 | 55 | 56 | <0.001 |
| Fibrates (%) | 4 | 1 | 3 | 5 | 0.247 |
| Ezetimib (%) | 6 | 1 | 2 | 2 | 0.008 |
| EPAs (%) | 7 | 3 | 1 | 4 | 0.017 |
| UA lowering agents (%) | 7 | 10 | 15 | 11 | 0.051 |
| Anti-platelet agents (%) | 20 | 24 | 25 | 32 | 0.339 |
| **Abbreviations:** ACEIs, angiotensin converting enzyme inhibitors; ARBs, angiotensin receptor blockers, CCBs, calcium channel blockers; DPP4, dipeptidyl peptidase 4; EPA, eicosapentaenoic acid; GIs, glycosidase inhibitors; GLP1-RA, glucagon-like peptide-1 receptors agonist; SGLT2, sodium-glucose cotransporter 2; TZDs, thiazolidinediones; UA, uric acid.  *Chi-square test or Fisher's exact test | | | | | |

**Table S3. Clinical characteristics at baseline in the four categories of body composition classified using percent of body fat**

|  | **Normal**  **(n = 193)** | **Sarcopenia**  **(n = 169)** | **Obesity**  **(n = 269)** | **Sarcopenic Obesity**  **(n = 85)** | ***P value** |
| --- | --- | --- | --- | --- | --- |
| Age (years) | 64 ± 12 | 69 ± 11^†^ | 62 ± 13 | 70 ± 10^†^ | < 0.001 |
| Gender (%male) | 40.9 | 66.9 | 45.7 | 76.4 | < 0.001 |
| Body fat (%) | 31.1 ± 5.4 | 26.6 ± 6.4^†^ | 40.8 ± 5.3^†^ | 38.0 ± 4.4^†^ | < 0.001 |
| Android fat mass (kg) | 1.7 (1.4-2.4) | 1.2 (0.9-1.6) ^†^ | 2.9 (2.4-3.7) ^†^ | 2.3 (2.1-3.1) ^†^ | < 0.001 |
| Gynoid fat mass (kg) | 2.6 (2.3-3.1) | 2.0 (1.5-2.6) ^†^ | 3.9 (3.1-4.4) ^†^ | 3.0 (2.6-3.6) ^†^ | < 0.001 |
| A/G ratio | 0.66 ± 0.21 | 0.56 ± 0.20^†^ | 0.80 ± 0.18^†^ | 0.80 ± 0.17^†^ | < 0.001 |
| SMI (kg/m^2^) | 6.83 ± 0.95 | 5.75 ± 0.84^†^ | 7.45 ± 1.20^†^ | 5.90 ± 0.90^†^ | < 0.001 |
| Body mass index (kg/m^2^) | 24.2 ± 2.8 | 20.4 ± 2.5^†^ | 30.0 ± 5.0^†^ | 24.3 ± 3.0 | < 0.001 |
| Duration of diabetes (years) | 11.5 ± 10.2 | 11.7 ± 11.3 | 9.8 ± 8.3 | 14.5 ± 12.4^†^ | < 0.001 |
| History of CVD (%) | 17 | 23 | 22 | 31 | 0.091 |
| Current smoker (%) | 11 | 18 | 12 | 16 | 0.162 |
| SBP (mmHg) | 129 ± 20 | 127 ± 19 | 134 ± 19^†^ | 130 ± 17 | 0.001 |
| DBP (mmHg) | 74 ± 12 | 73 ± 12 | 79 ± 13^†^ | 76 ± 13 | < 0.001 |
| Log ACR (mg/gCr) | 1.69 ± 0.73 | 1.80 ± 0.77 | 1.76 ± 0.80 | 1.76 ± 0.73 | 0.604 |
| eGFR (ml/min/1.73m^2^) | 73.3 ± 22.7 | 75.6 ± 26.4 | 69.8 ± 22.4 | 70.2 ± 32.5 | 0.086 |
| PDR (%) | 12 | 6 | 11 | 12 | 0.212 |
| HbA1c (%) | 8.4 ± 2.1 | 8.2 ± 2.1 | 8.2 ± 1.5 | 8.2 ± 1.7 | 0.792 |
| HbA1c (mmol/mol) | 68.0 ± 22.3 | 66.4 ± 23.2 | 66.2. ± 16.5 | 66.5 ± 18.7 | 0.792 |
| Triglycerides (mmol/l) | 1.31 (0.79-1.77) | 1.12 (0.80-1.73) | 1.60 (1.05-2.22) ^†^ | 1.32 (1.11-1.92) | < 0.001 |
| HDL cholesterol (mmol/l) | 1.46 ± 0.49 | 1.54 ± 0.51 | 1.36 ± 0.36^†^ | 1.41 ± 0.51^†^ | 0.001 |
| LDL cholesterol (mmol/l) | 2.90 ± 0.92 | 2.77 ± 0.81 | 2.84 ± 0.83 | 2.83 ± 0.92 | 0.512 |
| AST (U/l) | 22 (16-25) | 21 (17-24) | 24 (18-34) ^†^ | 22 (18-31) | < 0.001 |
| ALT (U/l) | 19 (15-28) | 16 (13-23) | 26 (17-43) ^†^ | 22 (16-35) | < 0.001 |
| γ-GTP (U/l) | 24 (17-44) | 27 (20-48) | 34 (21-60) ^†^ | 34 (20-54) | 0.003 |
| UA (μmol/L) | 299 ± 87 | 295 ± 87 | 335 ± 88^†^ | 309 ± 80 | < 0.001 |
| **Abbreviations:** ACR, albumin-to-creatinine ratio; A/G, android to gynoid fat; ALT, alanine transaminase; AST, aspartate transaminase; CVD, cardiovascular disease; DBP, diastolic blood pressure; eGFR, estimated glomerular filtration ratio; GTP, glutamyl transpeptidase; HDL, high-density lipoprotein; LDL, low-density lipoprotein; PDR, proliferative diabetic retinopathy; SBP, systolic blood pressure; SMI, skeletal muscle index; UA, uric acid.  *P Value for difference among the four groups in percepts (Chi-square test or Fisher's exact test) or means (ANOVA).  †P <0.05 vs Normal patients by Tukey's test. | | | | | |

**Table S4. Medication at baseline in the four categories of body composition classified using percent of body fat**

|  | **Normal**  **(n = 193)** | **Sarcopenia**  **(n = 169)** | **Obesity**  **(n = 269)** | **Sarcopenic Obesity**  **(n = 85)** | ***P value** |
| --- | --- | --- | --- | --- | --- |
| Insulin (%) | 38 | 39 | 33 | 39 | 0.537 |
| Sulfonylureas (%) | 21 | 22 | 24 | 18 | 0.648 |
| Metoformins (%) | 24 | 15 | 42 | 28 | <0.001 |
| Alpha-GIs (%) | 15 | 16 | 10 | 12 | 0.186 |
| Glinides (%) | 4 | 4 | 3 | 4 | 0.963 |
| TZDs (%) | 6 | 4 | 10 | 4 | 0.020 |
| DPP4 inhibitors (%) | 32 | 33 | 36 | 40 | 0.493 |
| SGLT2 inhibitors (%) | 1 | 0 | 1 | 1 | 0.636 |
| GLP1-RAs (%) | 1 | 2 | 4 | 2 | 0.117 |
| ACEIs (%) | 3 | 2 | 4 | 4 | 0.689 |
| ARBs (%) | 43 | 32 | 54 | 49 | <0.001 |
| CCBs (%) | 32 | 27 | 47 | 44 | <0.001 |
| Alpha blockers (%) | 2 | 1 | 3 | 0 | 0.256 |
| Beta blockers (%) | 8 | 7 | 13 | 20 | 0.008 |
| Diuretics (%) | 7 | 27 | 10 | 12 | 0.629 |
| Statins (%) | 41 | 28 | 59 | 45 | <0.001 |
| Fibrates (%) | 5 | 1 | 2 | 4 | 0.205 |
| Ezetimib (%) | 5 | 1 | 2 | 1 | 0.048 |
| EPAs (%) | 5 | 2 | 2 | 6 | 0.070 |
| UA lowering agents (%) | 9 | 7 | 14 | 15 | 0.065 |
| Anti-platelet agents (%) | 23 | 25 | 23 | 27 | 0.853 |
| **Abbreviations:** ACEIs, angiotensin converting enzyme inhibitors; ARBs, angiotensin receptor blockers, CCBs, calcium channel blockers; DPP4, dipeptidyl peptidase 4; EPA, eicosapentaenoic acid; GIs, glycosidase inhibitors; GLP1-RA, glucagon-like peptide-1 receptors agonist; SGLT2, sodium-glucose cotransporter 2; TZDs, thiazolidinediones; UA, uric acid.  *Chi-square test or Fisher's exact test | | | | | |

**Table S5. Clinical characteristics at baseline in the four categories of body composition classified using body mass index**

|  | **Normal**  **(n = 145)** | **Sarcopenia**  **(n = 221)** | **Obesity**  **(n = 317)** | **Sarcopenic Obesity**  **(n = 33)** | ***P value** |
| --- | --- | --- | --- | --- | --- |
| Age (years) | 64 ± 13 | 69 ± 11^†^ | 62 ± 12 | 69 ± 10^†^ | < 0.001 |
| Gender (%male) | 40.9 | 66.9 | 45.7 | 76.5 | < 0.001 |
| Body fat (%) | 31.6 ± 6.3 | 29.2 ± 7.5^†^ | 39.0 ± 6.3^†^ | 38.4 ± 6.6^†^ | < 0.001 |
| Android fat mass (kg) | 1.6 (1.3-2.3) | 1.4 (0.9-1.9) | 2.8 (2.3-3.6) ^†^ | 3.0 (2.4-3.5) ^†^ | < 0.001 |
| Gynoid fat mass (kg) | 2.6 (2.3-3.3) | 2.2 (1.7-2.5) ^†^ | 3.6 (2.7-4.2) ^†^ | 3.5 (2.9-3.9) ^†^ | < 0.001 |
| A/G ratio | 0.61 ± 0.21 | 0.62 ± 0.22 | 0.80 ± 0.18^†^ | 0.83 ± 0.16^†^ | < 0.001 |
| SMI (kg/m^2^) | 6.45 ± 0.80 | 5.70 ± 0.86 | 7.52 ± 1.11^†^ | 6.49 ± 0.48^†^ | < 0.001 |
| Body mass index (kg/m^2^) | 22.9 ± 1.9 | 20.9 ± 2.5^†^ | 29.7 ± 4.6^†^ | 27.2 ± 2.5^†^ | < 0.001 |
| Duration of diabetes (years) | 11.2 ± 10.8 | 12.4 ± 11.9 | 10.2 ± 8.3 | 14.1 ± 11.4^†^ | 0.029 |
| History of CVD (%) | 15 | 22 | 22 | 52 | 0.091 |
| Current smoker (%) | 13 | 18 | 11 | 18 | 0.162 |
| SBP (mmHg) | 129 ± 19 | 128 ± 19 | 134 ± 19^†^ | 128 ± 16 | 0.002 |
| DBP (mmHg) | 75 ± 12 | 74 ± 12 | 78 ± 13^†^ | 77 ± 14 | < 0.001 |
| Log ACR (mg/gCr) | 1.59 ± 0.70 | 1.80 ± 0.76^†^ | 1.79 ± 0.80^†^ | 1.66 ± 0.76 | 0.031 |
| eGFR (ml/min/1.73m^2^) | 75.2 ± 22.5 | 74.1 ± 28.9 | 69.4 ± 22.4 | 71.8 ± 27.2 | 0.055 |
| PDR (%) | 11 | 7 | 12 | 12 | 0.213 |
| HbA1c (%) | 8.4 ± 2.1 | 8.2 ± 2.0 | 8.2 ± 1.6 | 8.4 ± 1.6 | 0.816 |
| HbA1c (mmol/mol) | 67.9 ± 23.0 | 66.1 ± 22.3 | 66.5 ± 17.9 | 68.4 ± 17.4 | 0.816 |
| Triglycerides (mmol/l) | 1.39 (0.78-1.80) | 1.15 (0.81-1.72) | 1.47 (1.01-2.23) ^†^ | 1.29 (1.09-1.79) | < 0.001 |
| HDL cholesterol (mmol/l) | 1.50 ± 0.52 | 1.52 ± 0.51 | 1.35 ± 0.36^†^ | 1.34 ± 0.47 | < 0.001 |
| LDL cholesterol (mmol/l) | 2.94 ± 1.01 | 2.81 ± 0.86 | 2.83 ± 0.80 | 2.63 ± 0.69 | 0.240 |
| AST (U/l) | 22 (16-25) | 21 (17-23) | 23 (18-33) | 24 (19-31) ^†^ | 0.001 |
| ALT (U/l) | 20 (14-27) | 17 (13-25) | 25 (17-40) ^†^ | 22 (16-48) ^†^ | < 0.001 |
| γ-GTP (U/l) | 24 (17-43) | 29 (20-49) | 32 (22-57) | 35 (20-59) | 0.066 |
| UA (μmol/L) | 289 ± 86 | 297 ± 85 | 334 ± 87^†^ | 318 ± 86 | < 0.001 |
| **Abbreviations:** ACR, albumin-to-creatinine ratio; A/G, android to gynoid fat; ALT, alanine transaminase; AST, aspartate transaminase; CVD, cardiovascular disease; DBP, diastolic blood pressure; eGFR, estimated glomerular filtration ratio; GTP, glutamyl transpeptidase; HDL, high-density lipoprotein; LDL, low-density lipoprotein; PDR, proliferative diabetic retinopathy; SBP, systolic blood pressure; SMI, skeletal muscle index; UA, uric acid.  *P Value for difference among the four groups in percepts (Chi-square test or Fisher's exact test) or means (ANOVA).  †P <0.05 vs Normal patients by Tukey's test. | | | | | |

**Table S6. Medication at baseline in the four categories of body composition classified using body mass index**

|  | **Normal**  **(n = 145)** | **Sarcopenia**  **(n = 221)** | **Obesity**  **(n = 317)** | **Sarcopenic Obesity**  **(n = 33)** | ***P value** |
| --- | --- | --- | --- | --- | --- |
| Insulin (%) | 41 | 40 | 32 | 33 | 0.155 |
| Sulfonylureas (%) | 14 | 20 | 26 | 27 | 0.028 |
| Metoformins (%) | 19 | 15 | 42 | 49 | <0.001 |
| Alpha-GIs (%) | 11 | 15 | 12 | 12 | 0.710 |
| Glinides (%) | 6 | 4 | 3 | 6 | 0.481 |
| TZDs (%) | 4 | 3 | 11 | 6 | 0.003 |
| DPP4 inhibitors (%) | 29 | 33 | 37 | 55 | 0.030 |
| SGLT2 inhibitors (%) | 1 | 1 | 1 | 0 | 0.959 |
| GLP1-RAs (%) | 2 | 3 | 4 | 0 | 0.319 |
| ACEIs (%) | 3 | 2 | 5 | 6 | 0.445 |
| ARBs (%) | 35 | 35 | 56 | 58 | <0.001 |
| CCBs (%) | 30 | 30 | 45 | 52 | <0.001 |
| Alpha blockers (%) | 1 | 1 | 3 | 0 | 0.119 |
| Beta blockers (%) | 7 | 9 | 13 | 30 | 0.001 |
| Diuretics (%) | 6 | 10 | 10 | 15 | 0.360 |
| Statins (%) | 43 | 30 | 55 | 55 | <0.001 |
| Fibrates (%) | 4 | 1 | 3 | 6 | 0.268 |
| Ezetimib (%) | 6 | 1 | 2 | 3 | 0.050 |
| EPAs (%) | 6 | 3 | 2 | 3 | 0.113 |
| UA lowering agents (%) | 6 | 10 | 15 | 9 | 0.018 |
| Anti-platelet agents (%) | 19 | 23 | 25 | 46 | 0.012 |
| **Abbreviations:** ACEIs, angiotensin converting enzyme inhibitors; ARBs, angiotensin receptor blockers, CCBs, calcium channel blockers; DPP4, dipeptidyl peptidase 4; EPA, eicosapentaenoic acid; GIs, glycosidase inhibitors; GLP1-RA, glucagon-like peptide-1 receptors agonist; SGLT2, sodium-glucose cotransporter 2; TZDs, thiazolidinediones; UA, uric acid.  *Chi-square test or Fisher's exact test | | | | | |
